# Mutations in the C1 element of the insulin promoter lead to diabetic phenotypes in homozygous mice

**DOI:** 10.1038/s42003-020-1040-z

**Published:** 2020-06-16

**Authors:** Hirofumi Noguchi, Chika Miyagi-Shiohira, Yoshiki Nakashima, Takao Kinjo, Issei Saitoh, Masami Watanabe

**Affiliations:** 1 Department of Regenerative Medicine, Graduate School of Medicine, University of the Ryukyus, Okinawa, 903-0215 Japan; 2 Department of Basic Laboratory Sciences, School of Health Sciences, Faculty of Medicine, University of the Ryukyus, Okinawa, 903-0215 Japan; 3 Division of Pediatric Dentistry, Graduate School of Medical and Dental Science, Niigata University, Niigata, 951-8514 Japan; 4 Department of Urology, Okayama University Graduate School of Medicine, Dentistry and Pharmaceutical Sciences, Okayama, 700-8558 Japan

**Keywords:** CRISPR-Cas9 genome editing, CRISPR-Cas systems

## Abstract

Genome editing technologies such as CRISPR–Cas9 are widely used to establish causal associations between mutations and phenotypes. However, CRISPR–Cas9 is rarely used to analyze promoter regions. The insulin promoter region (approximately 1,000 bp) directs β cell-specific expression of insulin, which in vitro studies show is regulated by ubiquitous, as well as pancreatic, β cell-specific transcription factors. However, we are unaware of any confirmatory in vivo studies. Here, we used CRISPR–Cas9 technology to generate mice with mutations in the promoter regions of the insulin I (*Ins1*) and II (*Ins2*) genes. We generated 4 homozygous diabetic mice with 2 distinct mutations in the highly conserved C1 elements in each of the *Ins1* and *Ins2* promoters (3 deletions and 1 replacement in total). Remarkably, all mice with homozygous or heterozygous mutations in other loci were not diabetic. Thus, the C1 element in mice is required for *Ins* transcription in vivo.

## Abstract (web summary)

Noguchi et al. use CRISPR–Cas9 to generate mice with mutations in the promoter regions of the insulin I and II genes and show that only homozygous mutations in the highly conserved C1 element triggered a diabetic phenotype, highlighting the importance of this loci in insulin transcription.

## Introduction

The promoter of the gene encoding insulin comprises sequences that are immediately upstream of the transcription initiation site^1–5^. The insulin gene is specifically expressed in pancreatic β cells^4–6^, and its promoter regulates the rate of transcription in response to physiological regulators^7^. In the insulin promoter, a region of ~350 bp can direct β cell-specific expression of insulin, which is regulated by ubiquitous as well as pancreatic β cell-specific transcription factors^8^. In β cells, insulin gene (*Ins*) expression is controlled by multiple regulatory elements in the basal insulin promoter, which include the A, C and E elements, and three highly conserved enhancer elements in the human insulin promoter: A3 (bases –225 to –220)^9–12^, A2–C1 (bases –149 to –116)^13^ and E1 (bases –111 to–102)^14,15^. Certain transcription factors that govern β cell differentiation (pancreatic and duodenal homeobox1; Pdx1, neuronal differentiation; NeuroD and v-maf musculoaponeurotic fibrosarcoma oncogene family, protein A; MafA) interact directly with insulin promoter regulatory elements^9–15^. Most of these proteins bind to specific sites and form multiprotein transcriptional complexes situated on the insulin promoter^16,17^. The MafA and A2-binding factors cooperatively activate insulin gene expression^18^. However, these events were identified through in vitro studies, and we are unaware of comparable in vivo studies.

The use of clustered regularly interspaced short palindromic repeat (CRISPR)–Cas9 systems to manipulate mammalian genomes provides enormous opportunities for curing human diseases^19–22^. The nonhomologous end joining (NHEJ) mechanism leads to site-specific repair of the DNA break site, which causes different unpredictable insertions and deletions of various sizes. We took advantage of these mechanisms to develop mice in which the promoter regions of *Ins1* and *Ins2* were partially deleted. Although these features of the CRISPR–Cas9 system are disadvantageous for the generation of knockout mice with deletion of a single protein-encoding gene, the system is advantageous for evaluating the functions of promoter regions in vivo because multiple mice with different alterations of promoter sequences can be generated concurrently.

Here, we used CRISPR–Cas9 technology to generate mice with partial deletions of the *Ins1* and *Ins2* promoters, which showed that only homozygous mice with mutations in the highly conserved C1 elements of the *Ins1* and *Ins2* promoters developed diabetes, indicating the value of genome editing in studying the regulation of insulin synthesis in vivo.

## Results

### Construction of CRISPR–Cas9 expression vectors

**Fig. 1:**
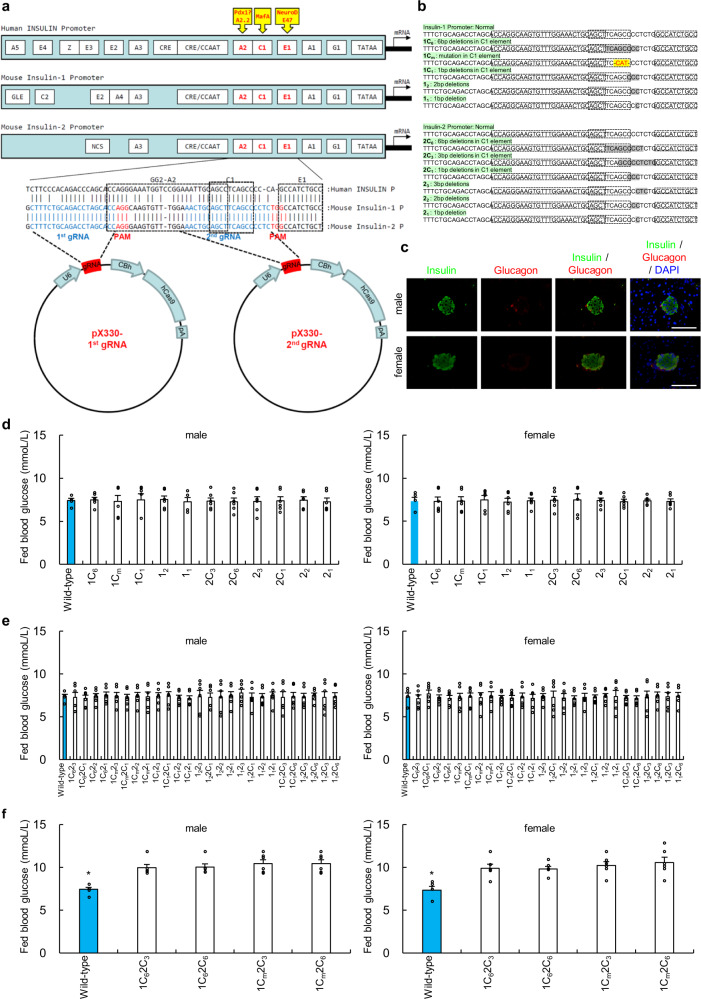
Construction of the CRISPR–Cas9 expression vectors and generation of mice with mutations of the insulin promoter. **a** Structures and sequences of the human and mouse insulin promoters and construction of the pX330-1st/2nd gRNA vectors. Bases –170 to –147 and –145 to –104 of the promoters of mouse *Ins1* and *Ins2* are identical, and bases –149 to –147 (AGG) and –114 to –112 (TGG) were used as the PAM sequences of the guide RNAs (gRNAs) for the CRISPR–Cas9 system. The 20-bp double-stranded DNAs (dsDNAs) derived from positions –169 to –150 and –134 to –115 of the *Ins1/2* promoters were inserted into pX330, and the resultant plasmids were designated pX330-1st gRNA and pX330-2nd gRNA, respectively. **b** Generation of mice with mutated insulin promoters. The mice with the four types of deletions and 1 replacement in the *Ins1* promoter and the six types of deletions in the *Ins2* promoter were generated. Sequences shaded in gray were deleted, and the yellow sequence indicates the replacement. **c** Immunohistochemical analysis of pancreatic islets (insulin, glucagon, and DAPI staining) from wild-type mice. Scale bars = 100 µm. **d** Blood glucose levels of fed wild-type (*n* = 6) and homozygous mice that had deletions or a replacement in only the *Ins1* promoter or a deletion in only the *Ins2* promoter (*n* = 6 each) at 12 weeks of age. **e** Blood glucose levels of fed wild-type (*n* = 6) homozygous mice that had any mutation in the *Ins1* promoter with ≤3-base deletions in the *Ins2* promoter (1C_6_2_3_, 1C_6_2C_1_, 1C_6_2_2_, 1C_6_2_1_, 1C_m_2_3_, 1C_m_2C_1_, 1C_m_2_2_, 1C_m_2_1_, 1C_1_2_3_, 1C_1_2C_1_, 1C_1_2_2_, 1C_1_2_1_, 1_2_2_3_, 1_2_2C_1_, 1_2_2_2_, 1_2_2_1_, 1_1_2_3_, 1_1_2C_1_, 1_1_2_2_, and 1_1_2_1_; *n* = 6 each) and ≤3-base deletions in the *Ins1* promoter with any deletion in the *Ins2* promoter (1C_1_2C_3_, 1C_1_2C_6_, 1_2_2C_3_, 1_2_2C_6_,1_1_2C_3_, and 1_1_2C_6_; *n* = 6 each) at 12 weeks of age. **f** Blood glucose levels of fed wild-type (*n* = 6) homozygous mice with >3-base deletions in the *Ins1* and *Ins2* promoters (1C_6_2C_3_, 1C_6_2C_6_, 1C_m_2C_3_, and 1C_m_2C_6_; n = 6 each) at 12 weeks of age. *p < 0.01 control vs. other groups. The error bars represent the standard error.

Despite the evolutionarily conserved function of insulin, the promoter and transcribed sequences of *Ins* are not well conserved between species^23,24^ (Fig. 1a, Supplementary Fig. 1). However, our detailed analysis of published mammalian promoter sequences revealed that most of the critical promoter sequence elements are well conserved^25^, particularly bases –151 to –103 of the mouse *Ins1* and *Ins2* promoters and bases –149 to –102 of the human insulin promoter (Fig. 1a, Supplementary Fig. 1). These sequences comprise the GG2–A2, C1 and E1 elements (Fig. 1a, Supplementary Fig. 1), which was previously known as the rat insulin promoter element 3 (RIPE3), to which cell-specific and ubiquitous factors bind^13^. For example, NeuroD binds the E1 element to form a heterodimer with the ubiquitously expressed bHLH factor E47. The C1 binding factor, which regulates pancreatic β cell-specific and glucose-regulated transcription of the insulin gene, is a member of the large family of Maf transcription factors^16–18,23–29^. The C1 element shares an overlapping DNA-binding region with the insulin enhancer element A2, and A2.2, a β cell-specific activator, binds to the overlapping A2 element^30^. Pdx1 binds to the GG2 element (bases –145 to –141 of the human insulin promoter^31^) (Supplementary Fig. 1).

We observed that bases –170 to –147 and –145 to –104 of the promoters of mouse *Ins1* and *Ins2* are identical and that bases –149 to –147 (AGG) and bases –114 to –112 (TGG) serve as protospacer adjacent motif (PAM) sequences in the guide RNA (gRNA) of the CRISPR–Cas9 system^19–21^. Therefore, we designed two gRNAs to target the promoter regions of mouse *Ins1* and *Ins2* (Fig. 1a). We used the pX330 vectors described in the “Methods” to express these gRNAs and Cas9 in mouse zygotes^32^.

### Generation of mice with insulin promoter mutations

To generate mice with insulin promoter mutations, pX330-1^st^/2^nd^ gRNA vectors were comicroinjected into the pronuclei of 310 one-cell-stage embryos obtained from C57BL/6J mice. Morphological abnormalities were undetectable in 252 (81.3%) of the embryos immediately after microinjection, which were consecutively transferred into the oviducts of pseudopregnant recipient the Institute for Cancer Research (ICR) mice to produce 86 neonates. Sequence analysis revealed that 23 mice had partial deletions and one replacement in either the *Ins1* or *Ins2* promoter or in both promoters and that 63 mice had normal or mosaic patterns. Mosaicism may be explained by mutations that occurred after the first or later embryonic divisions. Furthermore, Cas9 PCR products were not detected in 23 mice with deletions in either the *Ins1* or *Ins2* promoter or in both.

Among the 23 mice, we detected 5 and 6 types of mutations in the C1 elements or in the sequences bordering the C1 elements of the *Ins1* and *Ins2* promoters, respectively (Fig. 1b). To evaluate the importance of these promoter regions in vivo, the 11 mice with these mutations were crossed with wild-type C57BL/6J mice. Forty-one heterozygous mice with deletions in the *Ins1* promoter or *Ins2* promoter or in both were not diabetic (Supplementary Figs. 2, 3, Supplementary Table 1). The F1 mice were intercrossed to obtain homozygous F2 mice that had deletions in only the *Ins1* promoter or the *Ins2* promoter. These mice were not diabetic (Fig. 1d).

Thirty homozygous mice with mutations in both the *Ins1* and *Ins2* promoters were obtained by crossing the F3 and F4 mice (Supplementary Table 2). Homozygous mice with ≤3-base deletions in the *Ins1* promoter and any deletion in the *Ins2* promoter were not diabetic (Fig. 1e). Homozygous mice with any deletion in the *Ins1* promoter and ≤3-base deletions in the *Ins2* promoter were not diabetic (Fig. 1e). The most significant outcome of these studies was that the four types of mice with homozygous genotypes (1C_6_2C_6_, 1C_m_2C_6_, 1C_6_2C_3_, and 1C_m_2C_3_ mice) with >3-base deletions in both the *Ins1* and *Ins2* promoters were diabetic (Fig. 1f).

**Fig. 2:**
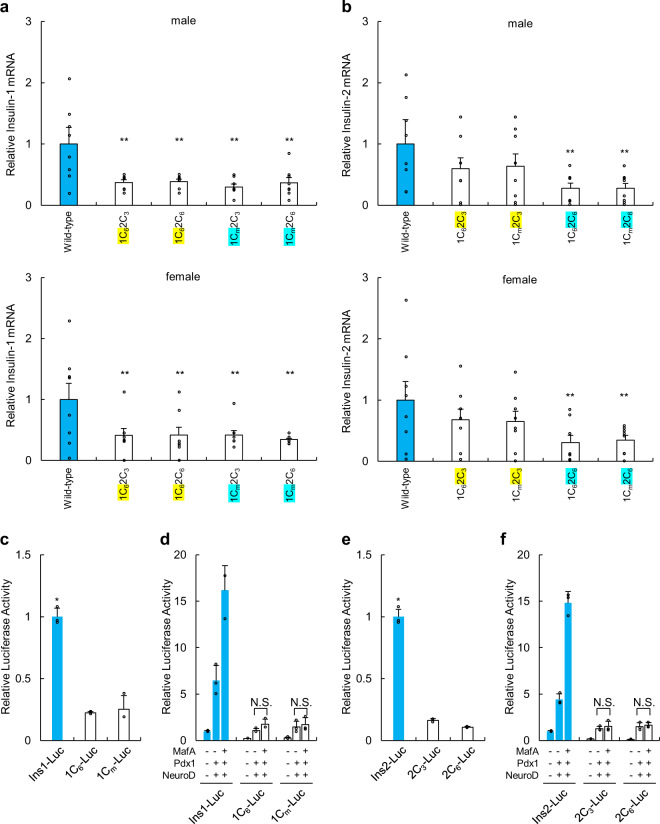
*Ins1* and *Ins2* mRNA levels and insulin promoter activities of mice with mutations in the *Ins1* and *Ins2* promoters. **a**, **b** qRT-PCR analysis of *Ins1* (**a**) and *Ins2* (**b**) in the pancreatic islets of mice with mutations of the insulin promoter. Pancreatic islets (purity > 95%) of wild-type mice served as a control. The data are expressed as the target gene-to-*Gapdh* ratio, and that of the control cells was arbitrarily defined as 1 (*n* = 8). **p < 0.05 control vs. other groups **c** Mouse *Ins1* promoter-luciferase plasmid containing ~1000 bp of the 5′-flanking sequences of the wild-type or mutated *Ins1* promoter region was transfected into βTC6 cells (β cell line). Forty-eight hours after transfection, cells were harvested and assayed (*n* = 3). *p < 0.01 control vs. other groups **d** Mouse *Ins1* promoter-luciferase plasmid containing approximately 1000 bp of the 5′-flanking sequences of the wild-type or mutated *Ins1* promoter region was cotransfected with combinations of the expression plasmids for MafA, Pdx1, and NeuroD into βTC6 cells (β cell line). Forty-eight hours after transfection, cells were harvested and assayed (*n* = 3). N.S. no significant differences. **e** Mouse *Ins2* promoter-luciferase plasmid containing approximately 1000-bp of the 5′-flanking sequences of the wild-type or *Ins2* deletion-containing promoter region was transfected into βTC6 cells (β cell line). Forty-eight hours after transfection, cells were harvested and assayed (*n* = 3). *p < 0.01 control vs. other groups **f** Mouse *Ins2* promoter-luciferase plasmid containing ~1000 bp of the 5′-flanking sequences of the wild-type or mutated *Ins2* promoter region was cotransfected with different combinations of the expression plasmids for MafA, Pdx1, and NeuroD into βTC6 cells (β cell line). Forty-eight hours after transfection, cells were harvested and assayed (*n* = 3). N.S. no significant differences. The error bars represent the standard error.

Quantitative RT-PCR analysis of mRNAs extracted from the pancreatic islets of the 4 types of mice (fasted) with diabetes showed that the mRNA levels of *Ins1* and *Ins2* were decreased compared with those of normal mice (fasted) (Fig. 2a, b). There were no significant differences in the levels of the *Pdx1*, *NeuroD*, and *MafA* mRNAs (Supplementary Fig. 4). Moreover, insulin promoter activity was significantly decreased for the two types of *Ins1* and *Ins2* promoters (Fig. 2c–f). MafA, Pdx1, and NeuroD synergistically activated the wild-type promoters. Mutations in C1 result in a sharp decrease in synergistic activation by MafA, Pdx1, and NeuroD. In particular, MafA did not induce the activation of promoters with C1 mutations (Fig. 2d, f).

### Phenotypes of 1C_6_2C_6_ mice

**Fig. 3:**
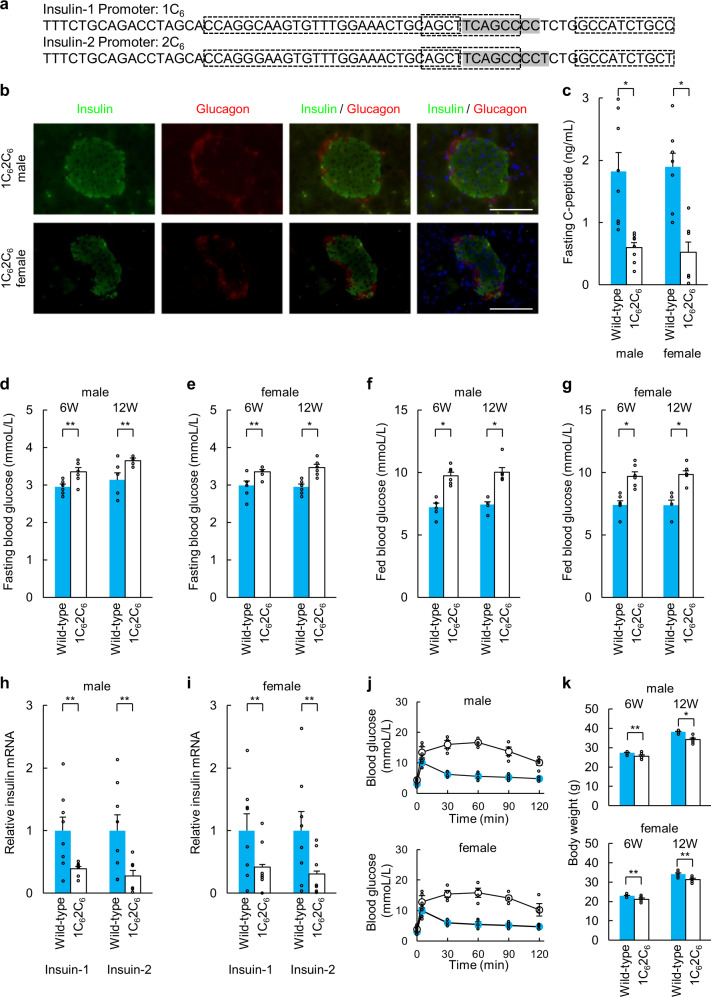
Phenotypes of 1C_6_2C_6_ mice. **a** Sequences of the *Ins1* and *Ins2* promoters in 1C_6_2C_6_ mice. **b** Immunohistochemical analysis of pancreatic islets (insulin, glucagon, and DAPI staining) in 1C_6_2C_6_ mice. Scale bars = 100 µm. **c** Fasting C-peptide levels of wild-type (*n* = 8) and 1C_6_2C_6_ mice (*n* = 8) at 12 weeks of age. **d**, **e** Fasting blood glucose concentrations of wild-type (*n* = 6) and 1C_6_2C_6_ mice (*n* = 6) at 6 and 12 weeks of age ((**d**): male, (**e**): female). **f**, **g** Blood glucose levels of fed wild-type (*n* = 6) and 1C_6_2C_6_ mice (*n* = 6) at 6 and 12 weeks of age ((**f**): male, (**g**): female). **h**, **i** qRT-PCR analysis of *Ins1* and *Ins2* in pancreatic islets of 1C_6_2C_6_ mice. Pancreatic islets (purity > 95%) of wild-type mice served as a control ((**h**): male, (**i**): female). The data are expressed as the gene-to-*Gapdh* ratio; that of the control cells was arbitrarily defined as 1 (*n* = 8). **j** Intraperitoneal glucose tolerance testing of wild-type (blue: *n* = 6) and 1C_6_2C_6_ mice (white: *n* = 6) at 12 weeks of age. **k** Body weights of wild-type (*n* = 6) and 1C_6_2C_6_ mice (*n* = 6) at 6 and 12 weeks of age. The error bars represent the standard error. *p < 0.01, **p < 0.05.

1C_6_2C_6_ mice (Fig. 3a) have deletions in the C1 elements of the *Ins1* and *Ins2* promoters. Immunohistochemical analysis of the pancreatic tissue of 1C_6_2C_6_ mice showed reduced insulin expression in islets (Fig. 3b) compared with that in islets of wild-type C57BL/6 mice (Fig. 1c). Fasting C-peptide levels were significantly reduced in 1C_6_2C_6_ mice at 12 weeks of age compared with those in wild-type mice (Fig. 3c). Blood glucose levels of fasted and fed 1C_6_2C_6_ mice at 6 and 12 weeks of age were elevated compared with those of wild-type C57BL/6 mice (Fig. 3d–g). *Ins1* and *Ins2* mRNA levels in pancreatic islets of 1C_6_2C_6_ mice (fasted) were significantly decreased compared with those of wild-type C57BL/6 mice (fasted) (Fig. 3h, i). Glucose tolerance tests showed significantly elevated glucose levels in male and female 1C_6_2C_6_ mice (Fig. 3j). The 1C_6_2C_6_ mice lost body weight at 6 and 12 weeks (Fig. 3k). The fertility of 1C_6_2C_6_ female mice was significantly reduced (40.0%).

### Phenotypes of 1C_m_2C_6_, 1C_6_2C_3_, and 1C_m_2C_3_ mice

**Fig. 4:**
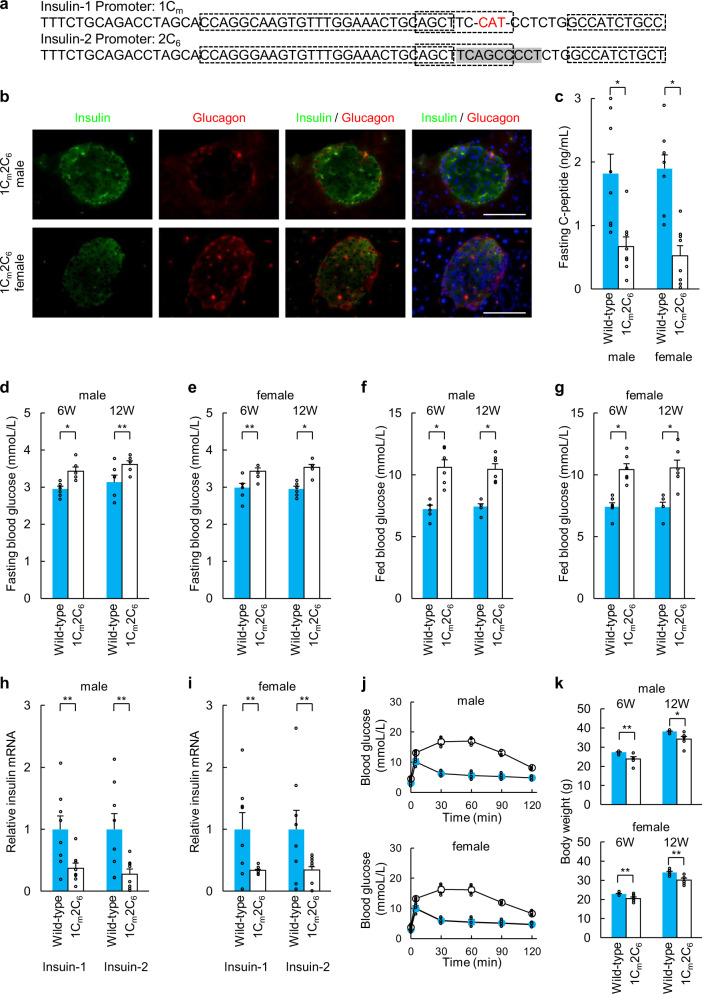
Phenotypes of 1C_m_2C_6_ mice. **a** Sequences of the *Ins1* and *Ins2* promoters in 1C_m_2C_6_ mice. **b** Immunohistochemical analysis of pancreatic islets (insulin, glucagon, and DAPI staining) in 1C_m_2C_6_ mice. Scale bars = 100 µm. **c** Fasting C-peptide levels of wild-type (*n* = 8) and 1C_m_2C_6_ mice (*n* = 8) at 12 weeks of age. **d**, **e** Fasting blood glucose concentrations of wild-type (*n* = 6) and 1C_m_2C_6_ mice (*n* = 6) at 6 and 12 weeks of age ((**d**): male, (**e**): female). **f**, **g** Blood glucose levels of fed wild-type (*n* = 6) and 1C_m_2C_6_ mice (*n* = 6) at 6 and 12 weeks of age ((**f**): male, (**g**): female). **h**, **i** qRT-PCR analysis of *Ins1* and *Ins2* in pancreatic islets of 1C_m_2C_6_ mice. Pancreatic islets (purity > 95%) of wild-type mice served as a control ((**h**): male, (**i**): female). The data are expressed as the gene-to-*Gapdh* ratio; that of the control cells was arbitrarily defined as 1 (*n* = 8). **j** Intraperitoneal glucose tolerance testing of wild-type (blue: *n* = 6) and 1C_m_2C_6_ mice (white: *n* = 6) at 12 weeks of age. **k** Body weights of wild-type (*n* = 6) and 1C_m_2C_6_ mice (*n* = 6) at 6 and 12 weeks of age. The error bars represent the standard error. *p < 0.01, **p < 0.05.

**Fig. 5:**
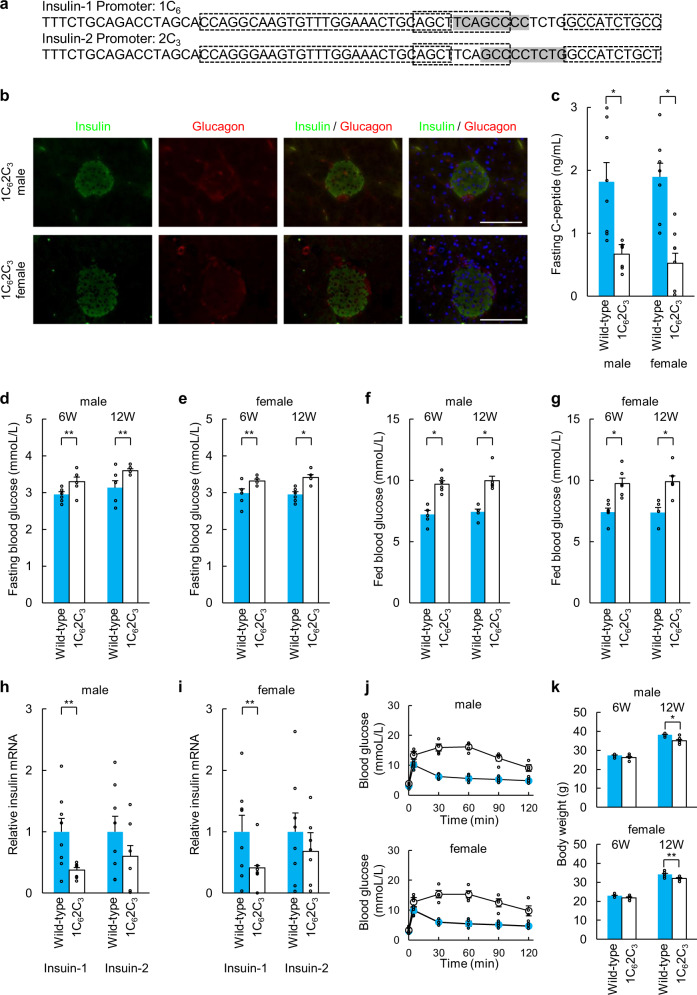
Phenotypes of 1C_6_2C_3_ mice. **a** Sequences of the *Ins1* and *Ins2* promoters in 1C_6_2C_3_ mice. **b** Immunohistochemical analysis of pancreatic islets (insulin, glucagon, and DAPI staining) in 1C_6_2C_3_ mice. Scale bars = 100 µm. **c** Fasting C-peptide levels of wild-type (*n* = 8) and 1C_6_2C_3_ mice (*n* = 8) at 12 weeks of age. **d**, **e** Blood glucose concentrations of fasting wild-type (*n* = 6) and 1C_6_2C_3_ mice (*n* = 6) at 6 and 12 weeks of age ((**d**): male, (**e**): female). **f**, **g** Blood glucose levels of fed of wild-type (*n* = 6) and 1C_6_2C_3_ mice (*n* = 6) at 6 and 12 weeks of age ((**f**): male, (**g**): female). **h**, **i** qRT-PCR analysis of *Ins1* and *Ins2* in pancreatic islets of 1C_6_2C_3_ mice. Pancreatic islets (purity > 95%) of wild-type mice served as a control ((**h**): male, (**i**): female). The data are expressed as the gene-to-*Gapdh* ratio; that of the control cells was arbitrarily defined as 1 (*n* = 8). **j** Intraperitoneal glucose tolerance testing of wild-type (blue: *n* = 6) and 1C_6_2C_3_ mice (white: *n* = 6) at 12 weeks of age. **k** Body weights of wild-type (*n* = 6) and 1C_6_2C_3_ mice (*n* = 6) at 6 and 12 weeks of age. The error bars represent the standard error. *p < 0.01, **p < 0.05.

**Fig. 6:**
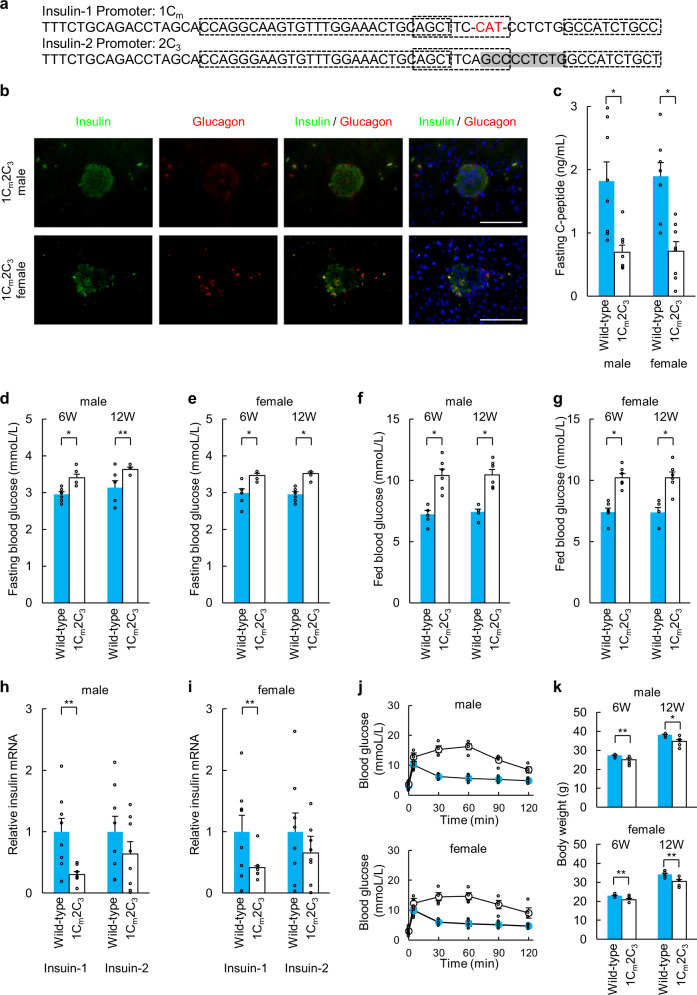
Phenotypes of 1C_m_2C_3_ mice. **a** Sequences of the *Ins1* and *Ins2* promoters in 1C_m_2C_3_ mice. **b** Immunohistochemical analysis of pancreatic islets (insulin, glucagon, and DAPI staining) in 1C_m_2C_3_ mice. Scale bars = 100 µm. **c** Fasting C-peptide levels of wild-type (*n* = 8) and 1C_m_2C_3_ mice (*n* = 8) at 12 weeks of age. **d**, **e** Blood glucose concentrations of fasting wild-type (*n* = 6) and 1C_m_2C_3_ mice (*n* = 6) at 6 and 12 weeks of age ((**d**): male, (**e**): female). **f** and **g** Blood glucose levels of fed wild-type (*n* = 6) and 1C_m_2C_3_ mice (*n* = 6) at 6 and 12 weeks of age ((**f**): male, (**g**): female). **h**, **i** qRT-PCR analysis of *Ins1* and *Ins2* in pancreatic islets of 1C_m_2C_3_ mice. Pancreatic islets (purity > 95%) of wild-type mice served as a control ((**h**): male, (**i**): female). The data are expressed as the gene-to-*Gapdh* ratio; that of the control cells was arbitrarily defined as 1 (*n* = 8). **j** Intraperitoneal glucose tolerance testing of wild-type (blue: *n* = 6) and 1C_m_2C_3_ mice (white: *n* = 6) at 12 weeks of age. **k** Body weights of wild-type (*n* = 6) and 1C_m_2C_3_ mice (*n* = 6) at 6 and 12 weeks of age. The error bars represent the standard error. *p < 0.01, **p < 0.05.

Analyses of the pancreatic tissues of 1C_m_2C_6_, 1C_6_2C_3_, and 1C_m_2C_3_ mice revealed similar alterations as those found in 1C_6_2C_6_ mice (Figs. 4a, b, 5a, b, 6a, b). The fasting C-peptide levels, fasting and fed blood glucose levels at 6 and 12 weeks of age, fasting *Ins1* mRNA levels in pancreatic islets, glucose tolerance test results, and body weights at 6 and 12 weeks of 1C_m_2C_6_, 1C_6_2C_3_, and 1C_m_2C_3_ mice were similar to those of 1C_6_2C_6_ mice and significantly different from those of wild-type C57BL/6 mice (Figs. 4c–g, j–k, 5c–g, j–k, 6c–g, j–k). In contrast, *Ins2* mRNA levels in the pancreatic islets of 1C_6_2C_3_ and 1C_m_2C_3_ mice were relatively but not significantly decreased compared with those of wild-type C57BL/6 mice (Figs. 5h, i, 6h, i), while *Ins2* mRNA levels in the pancreatic islets of 1C_6_2C_6_ mice were significantly decreased compared with those of wild-type C57BL/6 mice (Fig. 4h, i). The fertility of 1C_m_2C_6_, 1C_6_2C_3_, and 1C_m_2C_3_ female mice was significantly reduced and was similar to that of 1C_6_2C_6_ female mice.

## Discussion

Here, we used the CRISPR–Cas9 system to evaluate the functions of the promoter regions of *Ins1* and *Ins2* in vivo. We generated mice with 5 and 6 distinct deletions in the *Ins1* and *Ins2* promoters, respectively (Fig. 1b). Homozygous mice with deletions of ≤3 bases in the *Ins1* promoter with any deletion in the *Ins2* promoter (1C_1_*2any*, 1_2_*2any*, and 1_1_*2any*) or *Ins1* promoter and with deletions ≤3 bases of the *Ins2* promoter (*1any*2_3_, *1any*2C_1_, *1any*2_2_, and *1any*2_1_) were not diabetic (Fig. 1e, Supplementary Table 2). The 1–3 deleted bases were located between the C1 and E1 elements or within the sequences bordering the C1 element (Fig. 1b). Therefore, deletions of these 1–3 bases in the *Ins1/2* promoters did not affect insulin promoter activity. The other homozygous mice with deletions in both the *Ins1* and *Ins2* promoters were diabetic (Figs. 3–6). These data suggest that these sequences are important for insulin promoter activity in vivo. In vitro studies show that the C1 elements of the *Ins1* and *Ins2* promoters are required for *Ins* expression^13^. Furthermore, these sequences contain cell-specific and ubiquitous motifs that confer enhancer activity^13^, and proteins that bind to these sequences, such as MafA, show synergistic activity to activate *Ins* expression^17,18,31^. Our results provide compelling evidence that the sequences of the C1 elements in the *Ins1* and *Ins2* promoters are required for *Ins* expression in vivo.

Heterozygous mice with deletions in either the *Ins1* promoter or *Ins2* promoter or in both were not diabetic (Supplementary Figs. 2, 3, Supplementary Table 1). Moreover, homozygous mice with deletions in only the *Ins1* promoter or the *Ins2* promoter were not diabetic (Fig. 1d). It has been reported that mice with single homozygous null mutations of *Ins1* or *Ins2* were not diabetic because of compensatory responses, and there was a dramatic increase in *Ins1* transcripts in the *Ins2*^*−/−*^ mice^33^. Our data also showed that compensatory transcription of a functional insulin gene in homozygous mice with a deletion in the *Ins1* or *Ins2* promoter (Supplementary Fig. 3c, d) did not lead to diabetes.

In conclusion, we used the CRISPR–Cas9 system to analyze the insulin promoter by taking advantage of the site-specific repair mediated by NHEJ at the DNA break site, which leads to different unpredictable insertions or deletions of various sizes. Our findings show, to the best of our knowledge and for the first time, that the C1 elements of the insulin promoter in mice are required for its activity in vivo. More importantly, the CRISPR–Cas9 technique will provide a tool to generate knockout mice that can be used to evaluate promoter regions.

## Methods

### Generation of mice with insulin promoter mutations

To construct two Cas9–single-guide RNA (sgRNA) expression vectors, deoxyoligonucleotides (Fig. 1a: 1st gRNA and 2nd gRNA) were inserted into pX330 vectors^34^ (Addgene, Watertown, MA). Female C57BL/6J mice were injected with pregnant mare serum gonadotropin and human CG (hCG) at 48-h intervals and were then mated with male C57BL/6J mice. The fertilized one-cell embryos were collected from the oviducts. Subsequently, 5 ng/μL pX330-1st/2nd gRNA vectors were injected into the pronuclei of these one-cell embryos, which were transferred into pseudopregnant Institute of Cancer Research (ICR) mice. F0 mice were genotyped to detect the presence of mutations in the *Ins1/2* promoters. F0 mice were checked for the Cas9 transgene and for off-target effects. F0 mice were mated with C57BL/6N mice to obtain the F1 offspring.

The mutant mice and littermate wild-type mice were maintained on a C57BL/6 background. Mice were housed under a 12:12-h light/dark cycle in a room with controlled temperature and humidity. Food and water were provided without restrictions. The Institutional Animal Care and Use Committee of the University of the Ryukyus and the University of Tsukuba approved the animal studies.

### Construction of insulin promoter-luciferase plasmids

Wild-type or mutated insulin promoter cDNAs containing ~1000 bp of the 5′-flanking sequences of the *Ins1/2* promoter regions were amplified using PCR with appropriate linker-containing primers, which were then used to replace the promoter region of the Rat *Ins2* promoter-reporter (luciferase) plasmid^35^ using a ligation kit (TaKaRa, Tokyo, Japan).

### Gene transfection and luciferase assay

Mouse *Ins1/2* promoter-reporter (luciferase) plasmids (1.0 μg) containing ~1000 bp of the 5′-flanking sequences of the wild-type or mutated *Ins1/2* promoter region were cotransfected with a combination of plasmids (MafA, Pdx1, and NeuroD, 100 ng of each plasmid) into βTC6 cells (β cell line) (ATCC, Manassas, VA) using Lipofectamine (Thermo Fisher Scientific, Tokyo, Japan) according to the conditions recommended by the manufacturer. Forty-eight hours after transfection, the cells were harvested and assayed (Promega, Madison, WI).

### Immunohistochemistry

Cells were fixed with 4% paraformaldehyde in PBS. After blocking with 20% AquaBlock (EastCoast Bio, North Berwick, ME, USA) for 30 min at room temperature, the cells were incubated overnight at 4 °C with a guinea pig anti-insulin antibody (1:100; Abcam, Tokyo, Japan) or a mouse anti-glucagon antibody (1:200; Merck, Tokyo, Japan) and then for 1 h at room temperature with FITC-conjugated anti-guinea pig IgG (1:250; Abcam) or TRITC-conjugated anti-mouse IgG (1:250; Merck). Sections were treated with mounting medium to detect the fluorescence emitted by DAPI (Vector Laboratories, Peterborough, UK).

### Isolation of mouse pancreatic islets

Islets were isolated from the pancreata of normal and genome-edited mice^36,37^. For islet isolation, the common bile duct was cannulated and injected with cold Hank’s Balanced Salt Solution (HBSS, Thermo Fisher Scientific) containing 1.5 mg/ml collagenase (Roche Boehringer Mannheim, Indianapolis, IN). The islets were separated with a Histopaque 1077 (Merck) density gradient, hand-picked using a dissecting microscope to ensure pure islet preparation and used immediately.

### Quantitative RT-PCR

Total RNA was extracted from the isolated islets using an RNeasy Mini Kit (Qiagen, Tokyo, Japan). After quantifying the RNA using spectrophotometry, 2.5 µg of RNA was heated at 85 °C for 3 min and then reverse-transcribed in a 25-µl reaction mixture containing 200 units of Superscript II RNase H-RT (Thermo Fisher Scientific), 50 ng random hexamers (Thermo Fisher Scientific), 160 µmol/l dNTP and 10 nmol/l dithiothreitol. The reaction mixture was incubated for 10 min at 25 °C, 60 min at 42 °C and 10 min at 95 °C.

The mRNAs were quantitated using the TaqMan real-time PCR system according to the manufacturer’s instructions (Applied Biosystems, Foster City, CA, USA). PCR was performed for 40 cycles, and the reactions were incubated for 2 min at 50 °C and 10 min at 95 °C for the initial steps. In each cycle, denaturation was performed for 15 s at 95 °C, and annealing/extension was performed for 1 min at 60 °C. PCR was performed in 20 µl of solution using cDNAs synthesized from 1.11 ng of total RNA. The amount of mRNA was normalized by dividing the amount of the mRNA of interest by that of *Gapdh* mRNA. Primers specific for mouse *Ins1*, *Ins2*, *Pdx1*, *NeuroD*, *MafA*, and *Gapdh* were purchased as Assays-on-Demand Gene Expression Products (Applied Biosystems).

### ELISA

The mice were fasted overnight. C-peptide was measured with a C-peptide ELISA kit (FUJIFILM Wako Shibayagi, Gunma, Japan).

### Intraperitoneal glucose tolerance testing

Intraperitoneal glucose tolerance testing was performed on 6- and 12-week-old mice. The mice were fasted overnight, after which glucose (2.0 g/kg body weight) was intraperitoneally injected. The blood glucose levels were measured before injection and at 5, 30, 60, 90, and 120 min after injection.

### Statistics and reproducibility

Error bars indicate standard error of at least triplicate measurements. Student’s *t* test was used to compare two samples from independent groups using Microsoft Excel. Repeated measures ANOVA was performed to compare data among groups. The differences between the groups were considered to be significant when *p* < 0.05.

### Reporting summary

Further information on research design is available in the Nature Research Reporting Summary linked to this article.

## Supplementary information

Supplemental Information

Supplementary Data 1

Reporting Summary

Description of Additional Supplementary Files

## Data Availability

Raw data used to generate the figures can be found in Supplementary Data 1. Additional data that support the findings of this study are available from the corresponding author, H.N., upon reasonable request.
