# Supplementary material for: Mutations in the C1 element of the insulin promoter lead to diabetic phenotypes in homozygous mice
Source: Commun Biol. 2020 Jun 16;3:309. doi: 10.1038/s42003-020-1040-z (PMC7297962; doi:10.1038/s42003-020-1040-z)
Supplement: Supplementary file 1 — Supplemental Information [file 42003_2020_1040_MOESM1_ESM.pdf]

Supplemental Information

Mutations in the C1 element of the insulin promoter lead to diabetic phenotypes in homozygous mice

***Address correspondence to:**
Hirofumi Noguchi, MD, PhD
Department of Regenerative Medicine
Graduate School of Medicine, University of the Ryukyus
207 Uehara, Nishihara, Okinawa 903-0215, Japan.
Tel: +81-98-895-1696; Fax: +81-98-895-1684
E-mail: noguchih@med.u-ryukyu.ac.jp

Supplemental tables/figures count: tables 2 / figures 4

```
                                   SP1                             A5
Human INS P  GGGACAGCAGCGCAAAGAGCCCCGCC-CTGCAGCCTCCAGCTC--TCCTGGTCTAATGTGG -320
             |||||| ||     | |  ||   |  ||||||  ||| |     |  ||    ||  ||
Mouse Ins1 P GGGACATCAATATTAGGTCCCTAACAACTGCAGTTTCCTGGGGAATGATGTGGAAAA-TGC -305
             ||||||   ||| ||||||||   ||||||||  ||||||||||||||||||||||| |||
Mouse Ins2 P GGGACAAAGATACTAGGTCCC---CAACTGCAACTTCCTGGGGAATGATGTGGAAAAATGC -318

                              E4                           E3
Human INS P  ------AAAGTGGCCCAGGTGAGGGCTTTGCTCTCCTGGAGACATTTGCCCCCAGCTGTGAGCAG -261
                   ||||  |   |||   |  ||   || | |||| ||||  || |||| |||| || | |
Mouse Ins1 P TCAGCCAAAGATG---AAGAAGGTCTCAC-CT-T-CTGG-GACAA-TGTCCCCTGCTGGGAACTG -248
             |||||||| ||  ---||||| |  |||| |  |-||||- ||||-|||||||||||| ||||||
Mouse Ins2 P TCAGCCAAGGACA---AAGAAAGCATCACCCACT-CTGG-AACAA-TGTCCCCTGCTGTGAACTG -249

                            E2                                             CRE
Human INS P  GGACAGGTCTGGCCACCGGGCCCC-TGGTTAAGACTCTAATGACCCGCTGGTCCTGAGGAAGAGG -197
             |  ||  || ||||| | || |||-|  |||||||| |||| ||||   |   || ||  ||| |
Mouse Ins1 P GTTCA--TCAGGCCATCTGGTCCC-TTATTAAGACTATAATAACCCTAAGA--CTAAGT-AGATG -189
             |||||--|||||||||| || ||| || |||||||| |||| |||||| ||--||||||-||| |
Mouse Ins2 P GTTCA--TCAGGCCATCAGGGCCCCTTGTTAAGACTCTAATTACCCTAGGA--CTAAGT-AGAGG -189
                                                  A3
                                                     GG2          GG1
Human INS P  TGCTGACGACCAAGGAGATCTTCCCACAGACCCAGCACCAGGGAAATGGTCCGGAAATTGC -126
             || ||| | |||| |||  ||| |  |||||| ||||||||| || || |  ||||| |||
Mouse Ins1 P TGTTGATGTCCAATGAGTGCTTTCTGCAGACCTAGCACCAGGCAAGTGTT-TGGAAACTGC -129
             |||||| |||||||||| |||||||||||||||||||||||| |||||||-||||||||||
Mouse Ins2 P TGTTGACGTCCAATGAGCGCTTTCTGCAGACCTAGCACCAGGGAAGTGTT-TGGAAACTGC -129
                  CRE/CCAAT                                 A2

Human INS P  AGCCTCAGCCCC-CA-GCCATCTGCCGACCC-CCCCACCCC-AGGCCCTAATGGGCCAGG -70
             ||| |||||||| |  |||||||||| |||| |||||||   || || ||||||||||
Mouse Ins1 P AGCTTCAGCCCCTCTGGCCATCTGCCTACCCACCCCACCTGGAGACCTTAATGGGCCAAA -69
             |||||||||||||||||||||||||  ||| ||||||||||||| |||||||||| ||||
Mouse Ins2 P AGCTTCAGCCCCTCTGGCCATCTGCTGACCTACCCCACCTGGAGCCCTTAATGGGTCAAA -69
                 C1             E1                           A1

Human INS P  CGGCAGGGGTTGACAGGTAGGGGAGATGGG---CTCTGAGACTATAAAGCCAGCGGGGGCCCAG -9
             | |||  |     ||||  |  |||| | |   || || |||||||||||  | |||   ||||
Mouse Ins1 P CAGCAAAGTC---CAGGGGGCAGAGAGGAGGTACTTTG-GACTATAAAGCTGGTGGGCATCCAG -9
             ||||||||||---||||||||||||||||||| |||||-| |||||||| | ||||| | ||||
Mouse Ins2 P CAGCAAAGTC---CAGGGGGCAGAGAGGAGGTGCTTTG-GTCTATAAAGGTAGTGGGGACCCAG -9
                                    G1

Human INS P  CAGCCCTCAGCCCTCCAGGACAGGCTGC-ATCAGAAGAGGCCAT--CAAGCAGG
              | ||| |||||||    |||  |||   |||||| || |||||  ||||||||
Mouse Ins1 P TAACCCCCAGCCCTTAGTGACCAGCTATAATCAGA-GA-CCATCAGCAAGCAGG
             ||||| |||||||| ||||| | |||| ||||| |  | |||||||||||||||
Mouse Ins2 P TAACCACCAGCCCTAAGTGATCCGCTACAATCA-AAAA-CCATCAGCAAGCAGG
```

Supplementary Fig 1 Noguchi et al.

Supplementary Fig. 1 Sequences of human and mouse insulin promoters.

Although the insulin promoter sequence is not as well conserved between species as the transcribed sequences of the insulin gene, most of the functionally critical promoter sequence elements are well conserved. In particular, bases –151 to –103 of the promoters of mouse *Ins1* and *Ins2* and bases –149 to –102 of the human insulin promoter are highly conserved. These sequences include the GG2-A2, C1 and E1 elements.

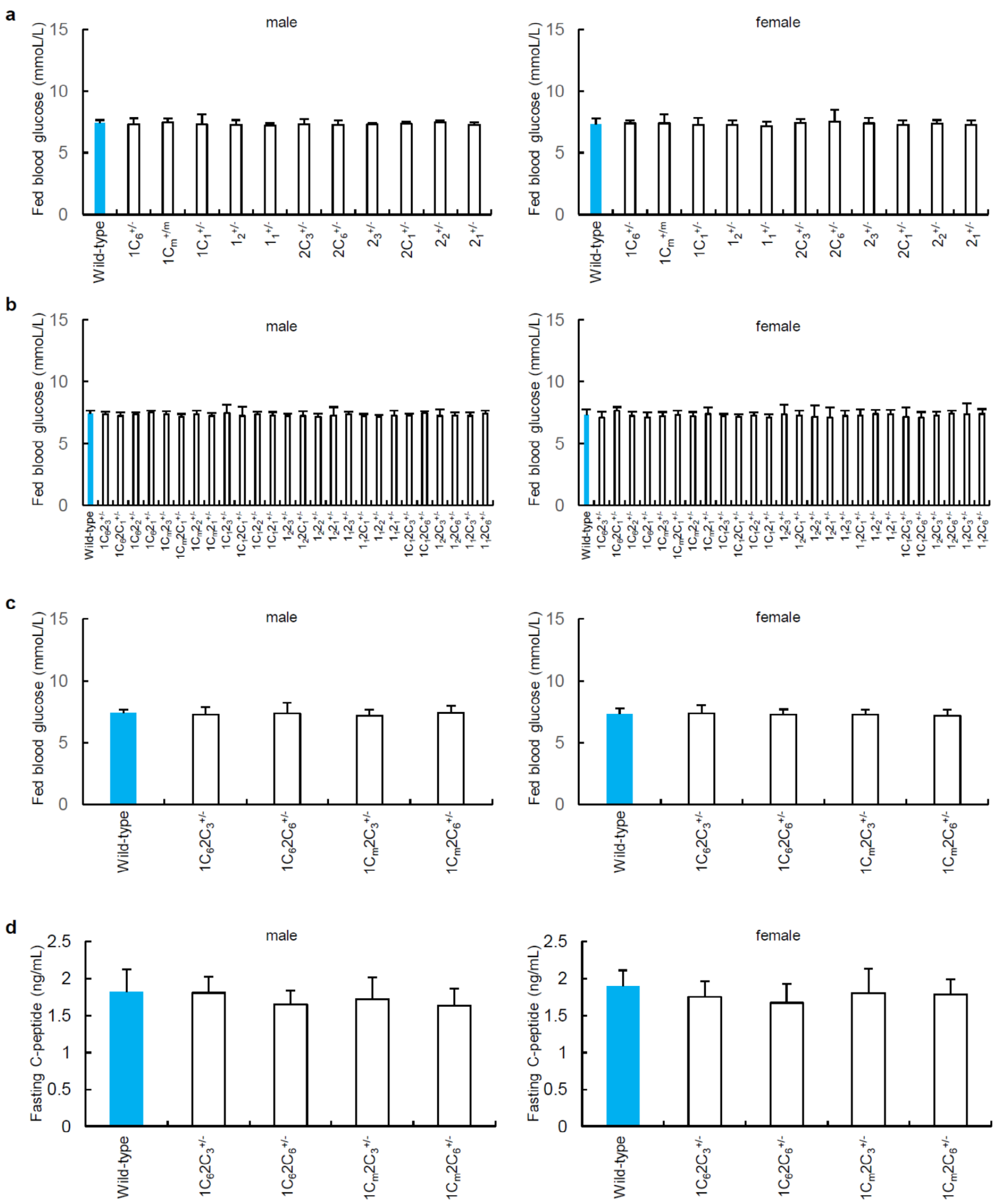

Supplementary Fig 2 Noguchi et al.

Supplementary Fig. 2 Blood glucose concentrations of heterozygous mice with mutations of the *Ins1* or *Ins2* promoters or both.

a Blood glucose levels of fed wild-type (n=6) and heterozygous mice with deletion or replacement of only the *Ins1* promoter or deletion of only the *Ins2* promoter (n=6 each) at 12 weeks of age. **b** Blood glucose levels of fed wild-type (n=6) and heterozygous mice with any deletion of the *Ins1* promoter along with ≤3-base deletions in the *Ins2* promoter ($1C_62_3^{+/-}$, $1C_62C_1^{+/-}$, $1C_62_2^{+/-}$, $1C_62_1^{+/-}$, $1C_m2_3^{+/-}$, $1C_m2C_1^{+/-}$, $1C_m2_2^{+/-}$, $1C_m2_1^{+/-}$, $1C_12_3^{+/-}$, $1C_12C_1^{+/-}$, $1C_12_2^{+/-}$, $1C_12_1^{+/-}$, $1_22_3^{+/-}$, $1_22C_1^{+/-}$, $1_22_2^{+/-}$, $1_22_1^{+/-}$, $1_12_3^{+/-}$, $1_12C_1^{+/-}$, $1_12_2^{+/-}$, and $1_12_1^{+/-}$; n=6 each) and ≤3-base deletions in the *Ins1* promoter with any deletion in the *Ins2* promoter ($1C_12C_3^{+/-}$, $1C_12C_6^{+/-}$, $1_22C_3^{+/-}$, $1_22C_6^{+/-}$, $1_12C_3^{+/-}$, and $1_12C_6^{+/-}$; n=6 each) at 12 weeks of age. **c** Blood glucose levels of fed wild-type (n=6) and heterozygous mutant mice with >3-base base deletions in the *Ins1* and *Ins2* promoters (n=6 each) at 12 weeks of age. **d** Fasting C-peptide levels of wild-type (n=8) and heterozygous mutant mice with >3-base base deletions in the *Ins1* and *Ins2* promoters (n=8 each) at 12 weeks of age.

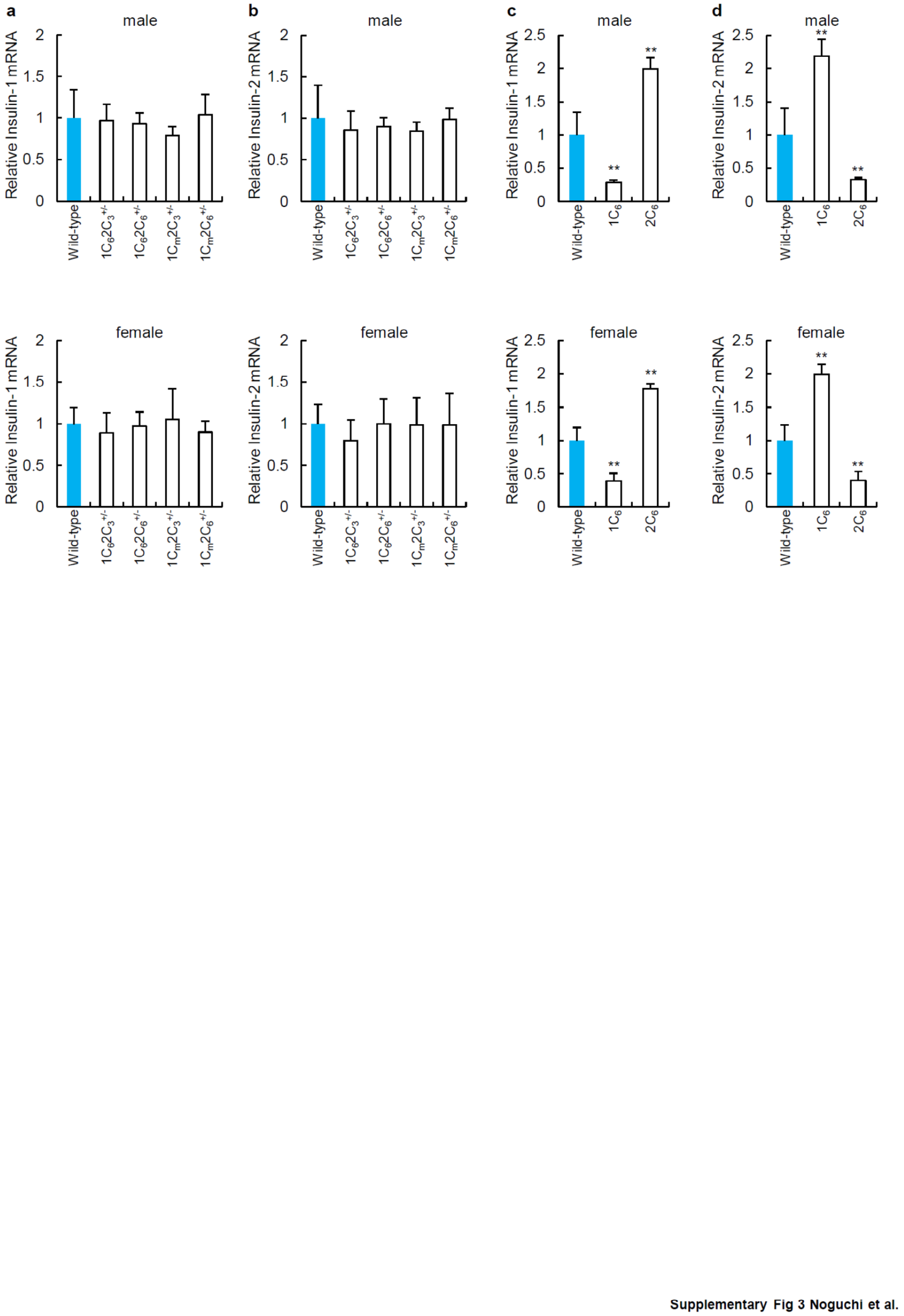

Supplementary Fig. 3. ***Ins1*** **and** ***Ins2*** **mRNA levels and insulin promoter activities**

of heterozygous/homozygous mice with mutations in the *Ins1* and *Ins2* promoters. **a and b** qRT-PCR analysis of *Ins1* (**a**) and *Ins2* (**b**) in pancreatic islets of heterozygous mice with mutations in the insulin promoter. **c and d** qRT-PCR analysis of *Ins1* (**c**) and *Ins2* (**d**) in pancreatic islets of homozygous mice with mutations in the insulin promoter. Pancreatic islets (purity >95%) of wild-type mice served as a control. The data are expressed as the target gene-to-*Gapdh* ratio; that of the control cells was arbitrarily defined as 1 (n=8). ** $p<0.05$

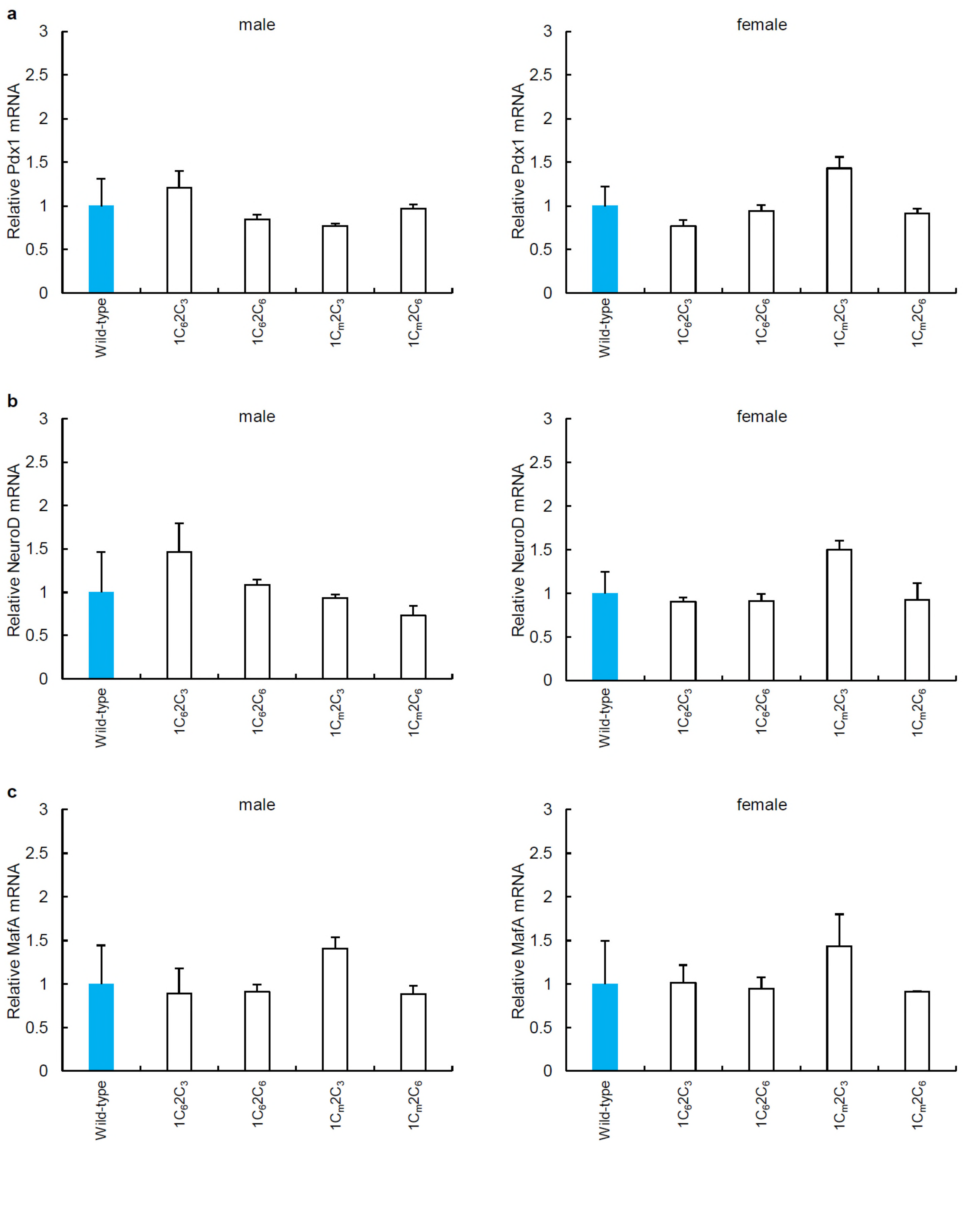

Supplementary Fig 4 Noguchi et al.

Supplementary Fig. 4 *Pdx1*, *NeuroD* and *MafA* mRNA levels in mice with insulin promoter mutations.

a qRT-PCR analysis of *Pdx1* expression in pancreatic islets of mice with mutations in the insulin promoter. **b** qRT-PCR analysis of *NeuroD* expression in pancreatic islets of mice with mutations in the insulin promoter. **c** qRT-PCR analysis of *MafA* expression in pancreatic islets of mice with mutations in the insulin promoter. Pancreatic islets (purity >95%) of wild-type mice served as a control. The data are expressed as the target gene-to-*Gapdh* ratio; that of the control cells was arbitrarily defined as 1 (n = 8). The error bars represent the standard error.

Supplementary Table 1. Heterozygous genotypes

Name	Mutated Elements in *Ins1* Promoter	Mutated Elements in *Ins2* Promoter	Diabetes	Figure #	Comments
$1C_62C_3^{+/-}$	C	C	-	Fig S2C	Mutation of elements bound by MafA in Ins1 and Ins2
$1C_62C_6^{+/-}$	C	C	-	Fig S2C	Deletion of elements bound by MafA in Ins1 and Ins2
$1C_62_3^{+/-}$	C	none	-	Fig S2B	
$1C_62C_1^{+/-}$	C	C (1 base only)	-	Fig S2B	
$1C_62_2^{+/-}$	C	none	-	Fig S2B	
$1C_62_1^{+/-}$	C	none	-	Fig S2B	
$1C_6^{+/-}$	C	none	-	Fig S2A	
$1C_m2C_3^{+/-}$	C (replacement)	C	-	Fig S2C	Mutation of elements bound by MafA in Ins1 and Ins2
$1C_m2C_6^{+/-}$	C (replacement)	C	-	Fig S2C	Mutation of elements bound by MafA in Ins1 and Ins2
$1C_m2_3^{+/-}$	C (replacement)	none	-	Fig S2B	
$1C_m2C_1^{+/-}$	C (replacement)	C (1 base only)	-	Fig S2B	
$1C_m2_2^{+/-}$	C (replacement)	none	-	Fig S2B	
$1C_m2_1^{+/-}$	C (replacement)	none	-	Fig S2B	
$1C_m^{+/-}$	C (replacement)	none	-	Fig S2A	
$1C_12C_3^{+/-}$	C (1 base only)	C	-	Fig S2B	
$1C_12C_6^{+/-}$	C (1 base only)	C	-	Fig S2B	
$1C_12_3^{+/-}$	C (1 base only)	none	-	Fig S2B	
$1C_12C_1^{+/-}$	C (1 base only)	C (1 base only)	-	Fig S2B	
$1C_12_2^{+/-}$	C (1 base only)	none	-	Fig S2B	
$1C_12_1^{+/-}$	C (1 base only)	none	-	Fig S2B	
$1C_1^{+/-}$	C (1 base only)	none	-	Fig S2A	
$1_22C_3^{+/-}$	none	C	-	Fig S2B	
$1_22C_6^{+/-}$	none	C	-	Fig S2B	

$1_2 2_3^{+/-}$	none	none	-	Fig S2B
$1_2 2C_1^{+/-}$	none	C (1 base only)	-	Fig S2B
$1_2 2_2^{+/-}$	none	none	-	Fig S2B
$1_2 2_1^{+/-}$	none	none	-	Fig S2B
$1_2^{+/-}$	none	none	-	Fig S2A
$1_1 2C_3^{+/-}$	none	C	-	Fig S2B
$1_1 2C_6^{+/-}$	none	C	-	Fig S2B
$1_1 2_3^{+/-}$	none	none	-	Fig S2B
$1_1 2C_1^{+/-}$	none	C (1 base only)	-	Fig S2B
$1_1 2_2^{+/-}$	none	none	-	Fig S2B
$1_1 2_1^{+/-}$	none	none	-	Fig S2B
$1_1^{+/-}$	none	none	-	Fig S2A
$2C_3^{+/-}$	none	C	-	Fig S2A
$2C_6^{+/-}$	none	C	-	Fig S2A
$2_3^{+/-}$	none	none	-	Fig S2A
$2C_1^{+/-}$	none	C (1 base only)	-	Fig S2A
$2_2^{+/-}$	none	none	-	Fig S2A
$2_1^{+/-}$	none	none	-	Fig S2A

Supplementary Table 2. Homozygous genotypes

Name	Mutated Elements in *Ins1* Promoter	Mutated Elements in *Ins2* Promoter	Diabetes	Figure #	Comments
$1C_62C_3$	C	C	+	Fig 1E, 2, 5, Fig S3	Deletion of elements bound by MafA in Ins1 and Ins2
$1C_62C_6$	C	C	+	Fig 1E, 2, 3, Fig S3	Deletion of elements bound by MafA in Ins1 and Ins2
$1C_62_3$	C	none	-	Fig 1D	
$1C_62C_1$	C	C (1 base only)	-	Fig 1D	
$1C_62_2$	C	none	-	Fig 1D	
$1C_62_1$	C	none	-	Fig 1D	
$1C_6$	C	none	-	Fig 1C	
$1C_m2C_3$	C (replacement)	C	+	Fig 1E, 2, 6, Fig S3	Mutation of elements bound by MafA in Ins1 and Ins2
$1C_m2C_6$	C (replacement)	C	+	Fig 1E, 2, 4, Fig S3	Mutation of elements bound by MafA in Ins1 and Ins2
$1C_m2_3$	C (replacement)	none	-	Fig 1D	
$1C_m2C_1$	C (replacement)	C (1 base only)	-	Fig 1D	
$1C_m2_2$	C (replacement)	none	-	Fig 1D	
$1C_m2_1$	C (replacement)	none	-	Fig 1D	
$1C_m$	C (replacement)	none	-	Fig 1C	
$1C_12C_3$	C (1 base only)	C	-	Fig 1D	
$1C_12C_6$	C (1 base only)	C	-	Fig 1D	
$1C_12_3$	C (1 base only)	none	-	Fig 1D	
$1C_12C_1$	C (1 base only)	C (1 base only)	-	Fig 1D	
$1C_12_2$	C (1 base only)	none	-	Fig 1D	
$1C_12_1$	C (1 base only)	none	-	Fig 1D	
$1C_1$	C (1 base only)	none	-	Fig 1C	
1_22C_3	none	C	-	Fig 1D	
1_22C_6	none	C	-	Fig 1D	

$1_2 2_3$	none	none	-	Fig 1D
$1_2 2C_1$	none	C (1 base only)	-	Fig 1D
$1_2 2_2$	none	none	-	Fig 1D
$1_2 2_1$	none	none	-	Fig 1D
1_2	none	none	-	Fig 1C
$1_1 2C_3$	none	C	-	Fig 1D
$1_1 2C_6$	none	C	-	Fig 1D
$1_1 2_3$	none	none	-	Fig 1D
$1_1 2C_1$	none	C (1 base only)	-	Fig 1D
$1_1 2_2$	none	none	-	Fig 1D
$1_1 2_1$	none	none	-	Fig 1D
1_1	none	none	-	Fig 1C
$2C_3$	none	C	-	Fig 1C
$2C_6$	none	C	-	Fig 1C
2_3	none	none	-	Fig 1C
$2C_1$	none	C (1 base only)	-	Fig 1C
2_2	none	none	-	Fig 1C
2_1	none	none	-	Fig 1C
