# Supplementary material for: Mutations in the C1 element of the insulin promoter lead to diabetic phenotypes in homozygous mice
Source: Commun Biol. 2020 Jun 16;3:309. doi: 10.1038/s42003-020-1040-z (PMC7297962; doi:10.1038/s42003-020-1040-z)
Supplement: Supplementary file 4 — Description of Additional Supplementary Files [file 42003_2020_1040_MOESM4_ESM.pdf]

Description of Additional Supplementary Files

File Name: **Supplementary Data 1**
Description: Raw data used to generate the charts present in the manuscript.
